# Infrared Diffusion‐Ordered Spectroscopy Reveals Molecular Size and Structure**

**DOI:** 10.1002/anie.202213424

**Published:** 2022-12-07

**Authors:** Giulia Giubertoni, Gijs Rombouts, Federico Caporaletti, Antoine Deblais, Rianne van Diest, Joost N. H. Reek, Daniel Bonn, Sander Woutersen

**Affiliations:** ^1^ Van't Hoff Institute for Molecular Sciences University of Amsterdam Science Park 904 1098XH Amsterdam The Netherlands; ^2^ Institute of Physics University of Amsterdam Science Park 904 1098XH Amsterdam The Netherlands

**Keywords:** Analytical Methods, Diffusion-Ordered Spectroscopy, Infrared Spectroscopy, Time-Resolved Spectroscopy, Two-Dimensional Infrared Spectroscopy

## Abstract

Inspired by ideas from NMR, we have developed Infrared Diffusion‐Ordered Spectroscopy (IR‐DOSY), which simultaneously characterizes molecular structure and size. We rely on the fact that the diffusion coefficient of a molecule is determined by its size through the Stokes–Einstein relation, and achieve sensitivity to the diffusion coefficient by creating a concentration gradient and tracking its equilibration in an IR‐frequency resolved manner. Analogous to NMR‐DOSY, a two‐dimensional IR‐DOSY spectrum has IR frequency along one axis and diffusion coefficient (or equivalently, size) along the other, so the chemical structure and the size of a compound are characterized simultaneously. In an IR‐DOSY spectrum of a mixture, molecules with different sizes are nicely separated into distinct sets of IR peaks. Extending this idea to higher dimensions, we also perform 3D‐IR‐DOSY, in which we combine the conformation sensitivity of femtosecond multi‐dimensional IR spectroscopy with size sensitivity.

## Graphical abstract

**Infrared diffusion‐ordered spectroscopy** is a simple and cost‐effective way to simultaneously characterize size and chemical structure of molecules, molecular aggregates/complexes, or small particles.

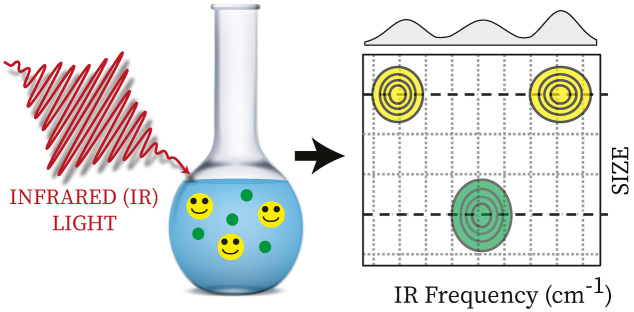

## Introduction

With the increased importance of self‐assembly in chemistry and biochemistry, size has become an increasingly important molecular parameter: besides polymers, one can think of supramolecular complexes, self‐assembling protein nanoparticles, and amyloids. With infrared (IR) spectroscopy, such complexes and aggregates are often difficult to distinguish from their monomeric building blocks: this is because IR spectroscopy is sensitive to the functional groups and conformation of a molecule,^[1]^ but generally not to its size. To address this issue, we have developed a method that simultaneously characterizes chemical structure and size: InfraRed Diffusion Ordered Spectroscopy (IR‐DOSY).

We took our inspiration from NMR Diffusion Ordered Spectroscopy,[^2^, ^3^, ^4^, ^5^, ^6^, ^7^, ^8^, ^9^, ^10^, ^11^, ^12^] in which different molecular species in a sample are separated based on their diffusion coefficient, resulting in a two‐dimensional NMR spectrum with chemical shift along one axis and diffusion coefficient along the other. The sensitivity to the diffusion coefficient is in this case achieved by a pulsed magnetic‐field gradient that creates a spatially varying phase shift in the spin precession, and the decay of this spatial variation due to diffusion of the spins is measured to determine the diffusion coefficient.^[2]^ Since the diffusion coefficient is inversely proportional to the size of a molecule or particle by the Stokes–Einstein relation,[^13^, ^14^] compounds with different sizes are nicely separated in a DOSY spectrum.

Just like NMR‐DOSY, IR‐DOSY also relies on the fact that the diffusion coefficient of a molecule (or particle) is determined completely by its size, but the diffusion‐coefficient selectivity is obtained in a different manner: instead of using a pulsed field gradient, we create a spatially inhomogeneous distribution of solute molecules using a flow method. The resulting two‐dimensional IR‐DOSY spectrum is similar to an NMR‐DOSY spectrum, but has IR frequency along the horizontal axis instead of the chemical shift. In an IR‐DOSY spectrum, one obtains the IR spectrum and the diffusion coefficient (or equivalently, size) of a compound at the same time; and mixtures of compounds with different sizes are nicely separated into distinct sets of peaks. We think IR‐DOSY can be a useful complement to NMR‐DOSY, for instance for paramagnetic compounds, compounds with strongly overlapping NMR spectra, or when the molecular tumbling is too slow for solution‐phase NMR.

## Results and Discussion

### Design and Operation Principle

**Figure 1:**
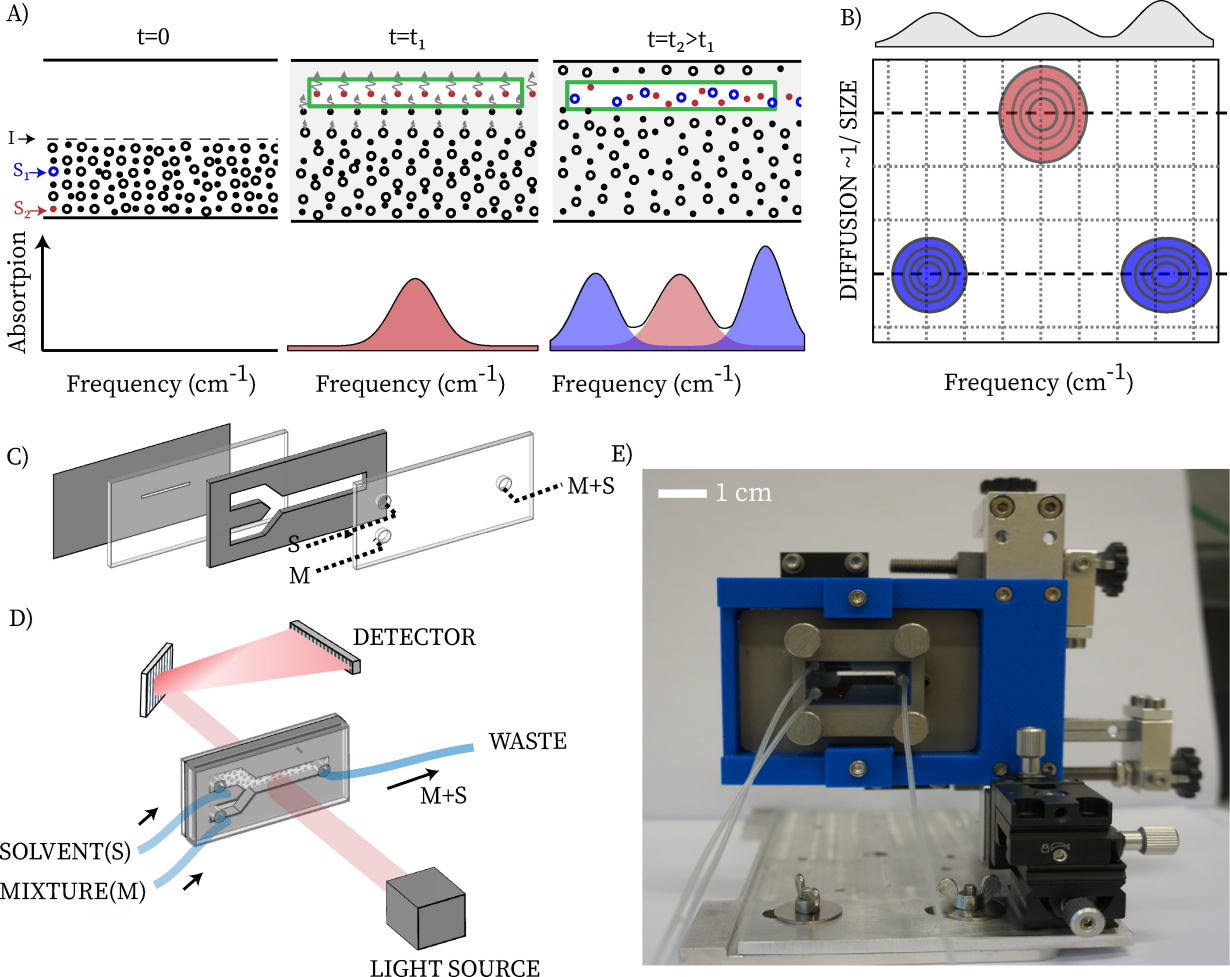
Infrared diffusion‐ordered spectroscopy. A) Operation principle: the sample solution (M) and pure solvent (S) are pumped into a channel (4 mm wide). The sample solution contains two molecular species, *S*_1_ and *S*_2_, with different sizes. The flow rates of the sample solution and solvent are the same, so the interface, *I*, between the two liquids is a line at the midpoint of the channel. When measuring the IR absorption at the end of the solvent‐filled half, a time‐dependent spectrum is observed, in which the absorption peaks of *S*_2_ appear before than the ones of *S*_1_ due to the higher diffusion coefficient of the smaller species. B) Schematic IR‐DOSY plot obtained from the time‐dependent data, in which the spectra of the different species are separated. C) Components of the designed IR‐DOSY. D) Schematic of experimental implementation of IR‐DOSY and E) IR‐DOSY sample cell in the lab.

Figure 1A shows how we simultaneously determine the diffusion coefficients and IR‐spectra of the species in a solution sample. In a thin space (typically 50–100 μm) between two IR‐transparent windows, we inject a solution and its solvent with the same flow rates. The flow rate is chosen such that the contact area between the liquids is a line in the middle of the channel (the flow is laminar, Reynolds number ≈0.02, see Supporting Information) and that the injection time through the channel is much shorter than the time required for diffusion of the solutes over the channel width. After we stop the flow, the solute molecules (large and small dots in the figure) start to diffuse into the pure solvent region, at a rate that depends on their diffusion coefficient. We measure the time‐dependent infrared absorption spectrum at a position in the sample space where there was initially only solvent. As time progresses, the solute molecules appear in the IR spectrum, at different rates depending on their size (small first, large later), and the time dependence of the amplitude of each species is given by the solution of the diffusion equation (see Methods section in the Supporting Information). From a global analysis of the time‐ and frequency‐dependent data we obtain an IR‐DOSY spectrum (Figure 1B). Figures 1C–E show the practical implementation. The IR‐DOSY setup consists of a home‐made liquid‐sample cell for IR transmission spectroscopy, combined with a standard double syringe pump to inject the solution and solvent. The cell fits into the standard sample‐cell mount of any IR spectrometer. The cell has two entrance holes and one exit hole. Using the syringe pump, we inject the sample solution (M) into one entrance, and pure solvent (S) into the other. After the pumping is stopped, the dissolved compounds diffuse into the solvent‐filled half of the cell, with a rate that depends on their size (see above). Since the IR beam in a standard FTIR spectrometer typically has a diameter of several mm, we ensure that we measure the IR spectrum of a specific region of the channel by means of an adjustable optical slit (green rectangle in Figure 1A).

### Real‐Time Imaging of the Experiment

**Figure 2:**
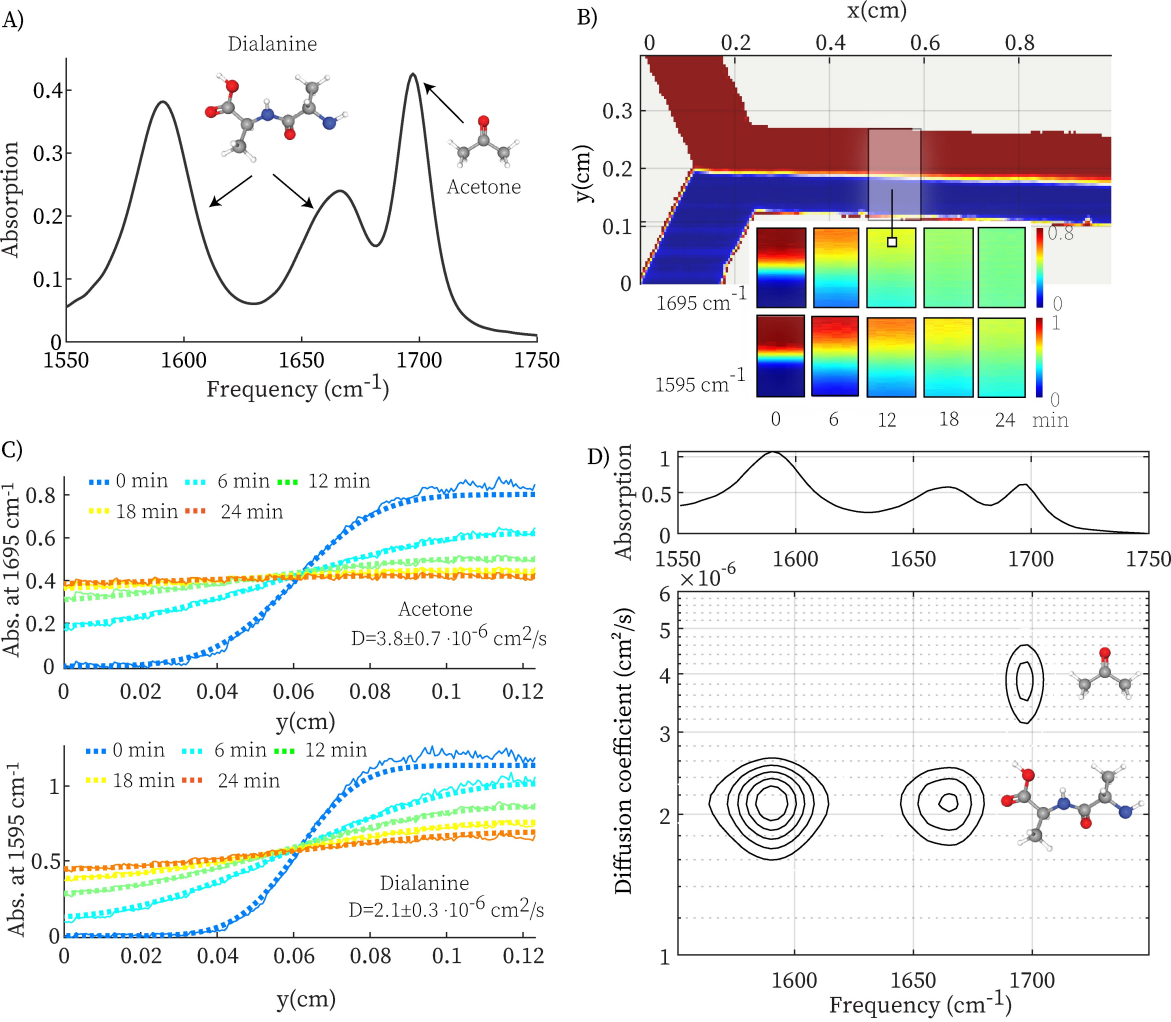
Imaging the IR‐DOSY device in operation. A) IR spectrum of the sample solution of acetone and dialanine in D_2_O. B) Top: IR‐microscope image of the channel while pumping the solution (bottom half) and solvent through the channel. Color bar: absorption at the frequency of the acetone (1695 cm^−1^). Bottom: close‐up images of the rectangle indicated in grey in the top panel, taken at different times after stopping the flow at *t*=0. The top row shows the absorption intensity at the absorption frequency of acetone, the bottom row at the frequency of dialanine (1695 and 1595 cm^−1^, respectively). C) Spatial distributions of the acetone and dialanine IR absorption at different time delays after stopping the flow. The dashed lines are least‐squares fits of the numerical solution of the diffusion equation. D) IR‐DOSY spectrum obtained from a global fit, see the text for details.

To investigate if the device works as predicted, we first use IR microscopy to image an experiment on a test sample in real time (in absence of the slit). As a sample, we use a solution of acetone and dialanine in D_2_O. In the initial experiments we added 2 % poly‐ethylene glycol (PEG) to ensure optimal flow, but we found that we also obtain stable flow in absence of PEG. In pure D_2_O, we obtain identical results, apart from slightly different diffusion coefficients, as expected from the Stokes–Einstein relation (see Supporting Information for the measurements in pure D_2_O). The IR spectrum (Figure 2A) of this solution contains bands at 1595, 1665 and 1700 cm^−1^, that are due to the carboxylate and amide groups of dialanine and the carbonyl group of acetone, respectively. Figure 2B shows the IR‐image of the entire channel (at the CO‐stretch frequency of acetone, 1695 cm^−1^) while pumping the sample solution and solvent through the channel. The liquid‐liquid contact is a straight line at the center of the rectangular channel, as expected since the flow‐injection rates of the two liquids are the same. The bottom panel of Figure 2B shows close ups of part of the channel (indicated by the rectangle in the top panel) at different time delays after stopping the flow at *t*=0. The top row shows the absorption at the IR‐frequency of acetone, the bottom row at the frequency of dialanine (1595 cm^−1^). The absorption is proportional to the species concentration, and we can clearly observe that acetone becomes evenly distributed on a faster time scale than dialanine, due to its higher diffusion coefficient.

Figure 2C shows the spatial distributions of the acetone and dialanine absorption across the channel at different times. These distributions can be well described using the solution of the diffusion equation (see Methods section in Supporting Information for the mathematical details), and from least‐squares fits (shown as the dashed lines in Figure 2C) we obtain the respective diffusion coefficients. We use these diffusion coefficients to globally fit the entire set of time‐ and frequency‐dependent absorption profiles in the 1550–1750 cm^−1^ frequency range. At each IR frequency, the time‐dependent absorption profile is the sum of that of acetone and dialanine, with weight factors determined by the absorption of each species at that IR frequency. We keep the value of the two diffusion coefficients of the two compounds fixed to the values obtained at their peak‐absorption frequencies, and treat the amplitudes of acetone and dialanine at each frequency as fit parameters. Similar to NMR‐DOSY,^[2]^ we use the values and uncertainties of the diffusion coefficients obtained from the fit together with the diffusion‐coefficient‐associated spectral amplitudes to obtain the IR‐DOSY plot shown in Figure 2D, in which the absorption peaks of acetone and dialanine are nicely separated.

### Infrared Diffusion‐Ordered Spectroscopy of Mixed Solutions

**Figure 3:**
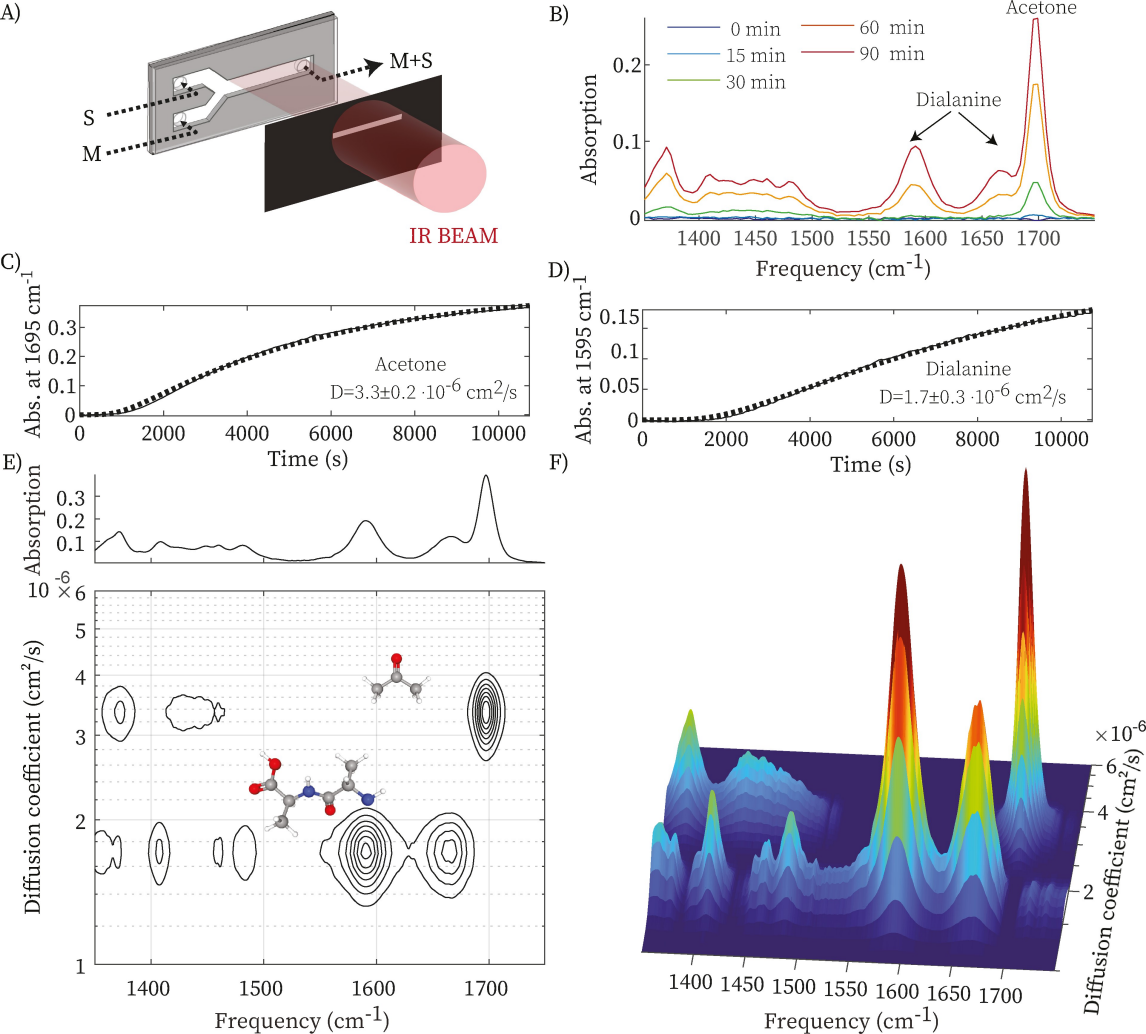
Infrared diffusion‐ordered spectroscopy of a double mixture using a conventional IR spectrometer. A) Schematic of the IR‐DOSY measurement using a slit to ensure spatial selectivity. B) Time‐dependent IR spectrum of a mixture containing acetone and dialanine. C)–D) Absorption at 1595 and 1695 cm^−1^ as a function of time after stopping the flow, respectively. E)–F) IR‐DOSY spectra of the double mix, showing which IR peak belongs to which compound.

After validating the operation principle, we now use the sample cell to obtain an IR‐DOSY spectrum with a conventional IR spectrometer in transmission mode. Since the infrared light beam in a typical IR spectrometer is comparable to the entire channel width (4 mm) of the cell, we use a slit to ensure spatial selectivity (Figure 3A). The slit is aligned parallel to the flow channel and positioned at the end of the solvent‐filled region, and has adjustable width to optimize the signal‐to‐noise ratio in case of low IR‐light intensity. Figures 3B–D show the results obtained with the sample of Figure 2 (a mixture of acetone and dialanine). Figure 3B shows the time‐dependent IR‐absorption spectrum recorded using the slit, at different times after stopping the flow. Since the slit blocks slightly more (10–30 %) IR light than a conventional spectrometer‐aperture, the signal‐to‐noise is slightly less than in a conventional FTIR spectrum. The IR bands of acetone appear faster than those of dialanine, and by least‐squares fitting the time‐ and frequency‐dependent data with the solution of the diffusion equation (see Methods section in the Supporting Information), we obtain the two diffusion coefficients and their associated species spectra. Figures 3E,F show the resulting two‐dimensional IR‐DOSY spectrum. The frequency region between 1350 and 1550 cm^−1^ is generally highly congested because of the overlap of many side‐chain modes, such as the CH‐bending modes. In the IR‐DOSY spectrum, we observe the spectral bands separated into two rows centered at *D*≈2×10^−6^ cm^2^ s^−1^ and *D*≈3.5×10^−6^ cm^2^ s^−1^. In the top row, the spectral bands are centered at 1695, 1450 and 1360 cm^−1^, which correspond to the absorption frequencies of acetone (see Figure S5 for the spectrum of a solution of only acetone). In the bottom row, we observe spectral bands at 1665, 1600, 1490, 1400 and 1360 cm^−1^, which correspond to the absorption frequencies of dialanine (see Figure S6). The IR‐DOSY spectrum neatly resolves the congested FTIR spectrum (top panel of Figure 3E) into the separate spectra of the two compounds, and the diffusion coefficients tells us their size.[^15^, ^16^] The separation into species spectra is especially useful in the fingerprint frequency region (below 1500 cm^−1^), which is very sensitive to molecular structure, but in the case of samples containing more than one compound tends to be congested when using conventional IR spectroscopy. Comparing the conventional and IR‐DOSY spectra (top and bottom panels in Figure 3E), it can be seen that in the latter the fingerprint modes of the two compounds are cleanly separated.

**Figure 4:**
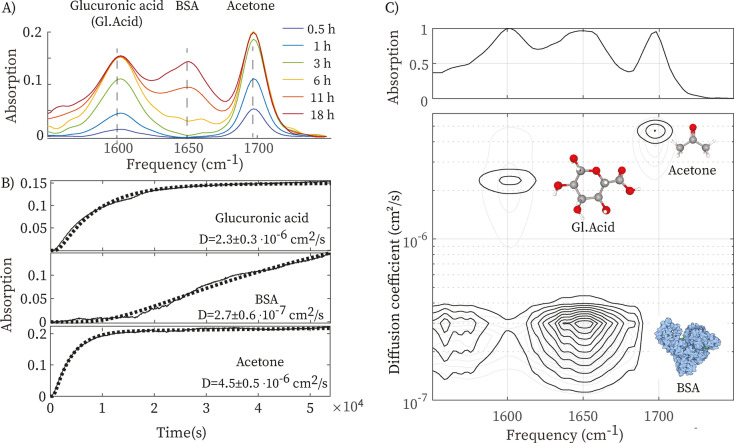
Infrared diffusion‐ordered spectroscopy of a triple mixture using a conventional IR spectrometer. A) Time‐dependent IR spectra of a mixture containing acetone, glucuronic acid and BSA. B) Absorption at 1595, 1650 and 1695 cm^−1^ as a function of time after stopping the flow. C) IR‐DOSY spectrum of the triple mix, showing which IR peak belongs to which compound. For comparison, we also plot the IR‐DOSY spectrum obtained for the same mixture using IR‐microscopy (grey contours).

Figure 4 shows the results obtained with a sample solution containing three compounds: acetone, glucuronic acid, and bovine serum albumin (BSA), a large protein diffusing significantly slower than the other two compounds. Figure 4A shows the time‐dependent absorption spectrum, in which the peaks of acetone (C=O stretch) and glucuronic acid (COO^−^ stretch) appear first, while the BSA amide‐I band appears much later. Figure 4B shows the absorption as a function of time at the peak‐frequencies of the three compounds. By fitting the data with the solution of the diffusion equation, we obtain the diffusion coefficients of the three compounds. Using these diffusion coefficients, we globally fit the full time‐ and frequency‐dependent data set. Figure 4C shows the IR‐DOSY spectrum obtained in this way. In the IR‐DOSY spectrum, the peaks are distributed in three different rows that are centered at three different diffusion coefficients (2.3±0.3×10^−6^, 2.7±0.6×10^−7^ and 4.5±0.5×10^−6^ cm^2^ s^−1^). In the bottom row, we observe two signatures at 1580 cm^−1^ and 1650 cm^−1^, which are due to the carboxylate and amide groups of BSA (see Figure S7). It is interesting to note that the carboxylate peak at 1580 cm^−1^ is difficult to distinguish in the conventional infrared spectrum of the mixture (top panel of Figure 4C) because it overlaps with the strong peak from glucuronic acid. The size difference between BSA and glucuronic acid (and the resulting difference in diffusion coefficient) ensures that in the IR‐DOSY spectrum these peaks are well separated.

In these examples, the number of molecular species in the sample was known beforehand, but just as in NMR‐DOSY, this is not a necessary requirement. If the number of overlapping peaks is not too large, then at each IR frequency the absorption is dominated by one molecular species, and so the time‐dependence of the absorption at that frequency can be fitted with a single‐component diffusion time‐profile, with the diffusion coefficient as the free parameter.^[2]^ The number of rows of peaks in the resulting DOSY spectrum then gives the number of species (more precisely, molecular sizes) present in the sample, and each row the corresponding species IR spectrum.

For larger molecules, the time for complete diffusion over the channel may take considerable time. However, the initial time‐dependence shows a lag phase that has a duration determined by the diffusion coefficient (see middle panel of Figure 4B), and so the measurement time can be much shorter than the time it takes for the largest molecules to completely diffuse over the channel width. Furthermore, by using a narrower channel and/or positioning the slit closer to the liquid‐liquid interface, the diffusion time can easily be reduced if necessary, because the characteristic diffusion time scales with the square of the channel width.^[14]^

### Femtosecond 3D‐IR‐DOSY

The above results show that with IR‐DOSY, any standard FTIR spectrometer can be used to disentangle the different species present in a mixture and obtain their separate IR spectra, provided the sizes are sufficiently different. Although FTIR spectroscopy is the most common infrared technique used to study molecules, much more detailed knowledge of molecular structure and conformation can be obtained by using two‐dimensional infrared (2D‐IR) spectroscopy (see refs. [17, 18, 19, 20, 21, 22, 23] for reviews on 2DIR spectroscopy). If there are several vibrational modes in a molecule, their couplings show up in the 2DIR spectrum as cross peaks, that are sensitive probes of the structure.[^17^, ^20^, ^22^] In particular, secondary structures of proteins are much more reliably identified by 2DIR, because they appear as specific spectral signatures in 2DIR spectra.[^17^, ^20^, ^22^] Furthermore, the contour shapes in 2DIR spectra provide direct information on the short‐time conformational heterogeneity of the molecules, and from the change in contour shape upon increasing the delay time between the two IR pulses, the correlation function of the conformational fluctuations is obtained.[^17^, ^24^] All these features can be combined with size selectivity by performing 2DIR spectroscopy in an IR‐DOSY sample cell.

**Figure 5:**
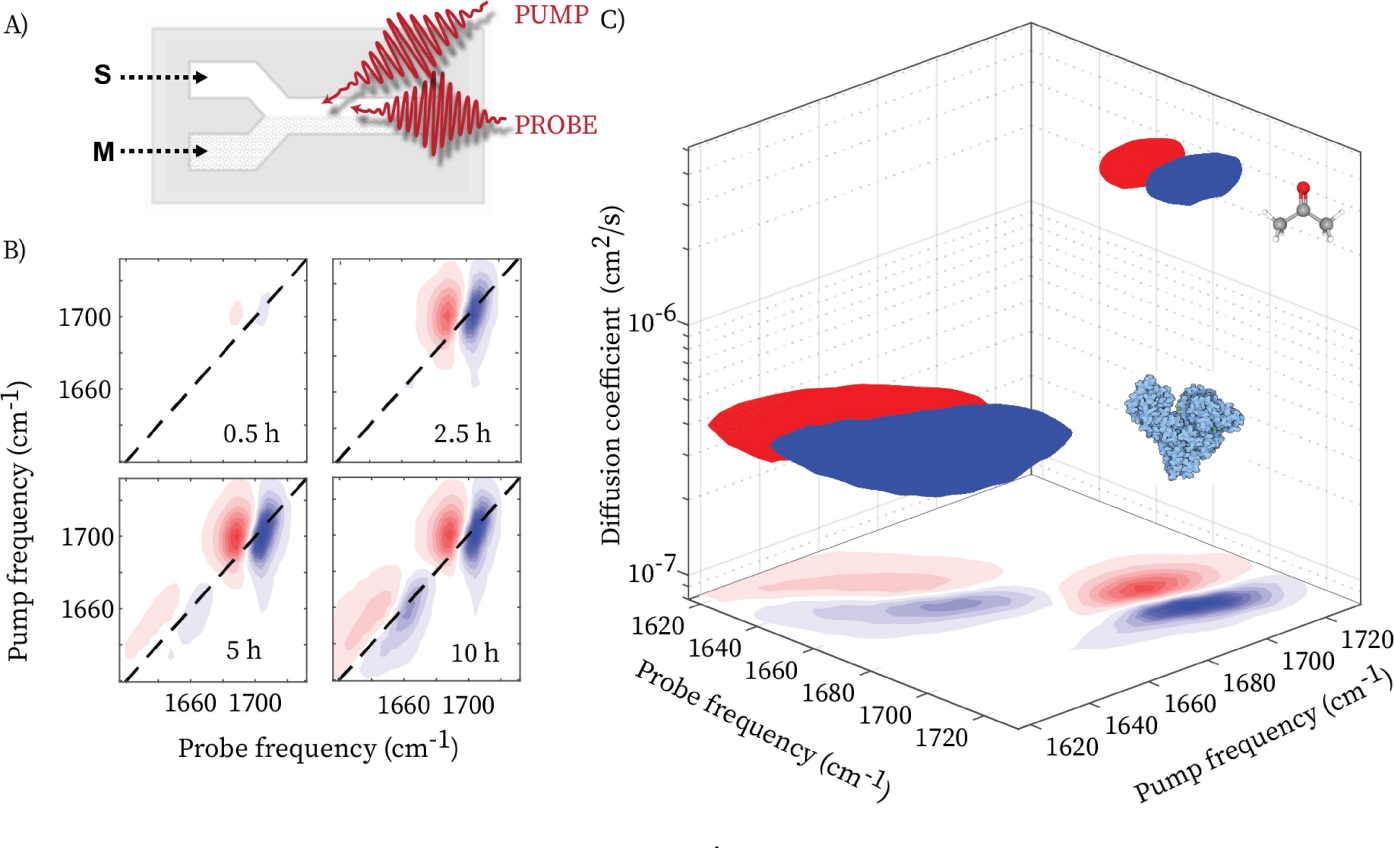
A) Schematic of 3D‐IR‐DOSY spectroscopy using a pump‐probe 2D‐IR setup. B) Time series of 2DIR spectra collected at different time delays after stopping the flow for a solution containing acetone and BSA. C) 3D‐IR‐DOSY spectrum obtained from the time‐resolved data.

To combine the enhanced structure sensitivity of 2D‐IR spectroscopy with molecular‐size selectivity, we perform 2D‐IR spectroscopy with the DOSY cell to obtain 3D‐IR‐DOSY spectra, somewhat similar to higher‐dimensional NMR‐DOSY.[^25^, ^26^, ^27^, ^28^] Figure 5 shows a schematic of the 3D‐IR‐DOSY experiment using pump‐probe 2D‐IR spectroscopy.^[17]^ Contrary to the IR‐DOSY measurement performed using a FTIR spectrometer, no slit is necessary to select part of the channel, since the IR pulses in 2DIR experiments are focused (in our setup the focal diameter is ≈250 μm). Figure 5B shows 2D‐IR spectra at increasing time delays after stopping the flow. In the 2D‐IR spectra, each vibrational mode of a molecule gives rise to a +/− doublet on the diagonal.^[17]^ The blue contours represent a decrease in absorption (Δ*A*<0) due to depletion of the *v*=0 state by the IR pump pulse, and the positive signal at lower probe frequency (red contours) is due to the induced absorption of the *v*=1→2 transition. In this particular sample there are no cross peaks, since acetone and BSA both have only one vibrational mode in this frequency region. With increasing time, first the 2D‐IR peaks of acetone appear (*v*_pump_=1700 cm^−1^), and at later times the 2D‐IR peaks of BSA (*v*_pump_=1660 cm^−1^). We fit the absorption profiles taken at the bleach position of the two pair of diagonal peaks to obtain the diffusion coefficients, and using these values, we globally fit the time‐dependent 2DIR spectra, obtaining a three‐dimensional IR‐DOSY spectrum (see Supporting Information for details). Similarly to the IR‐DOSY plots, we observe 3D‐spectral signatures at two different diffusion coefficients, 3.4±0.5×10^−6^ and 3.5±0.7×10^−7^ cm^2^ s^−1^. The 3D‐spectral signature at the lower diffusion coefficient consists of the 2D‐IR peaks of the BSA, while the one at the higher diffusion coefficient consists of the 2D‐IR peaks of the acetone. Comparing the tilt of the 3D peaks with respect to the pump‐frequency axes for BSA and acetone, it can immediately be seen that the former has not only larger size but also more structural heterogeneity than the latter.^[17]^ Similarly to IR‐DOSY, we thus demonstrate that 2D‐IR can be combined with molecular size‐selectivity to obtain 3D‐IR‐DOSY spectroscopy which separates the 2D‐IR spectral signatures of size‐selected species present in a mixture.

3D‐IR‐DOSY is a powerful analytical tool, opening up novel applications of 2D‐IR to study supramolecular complexes, self‐assembly protein fibers, and amyloids. Since recent work has demonstrated the potential of 2D‐IR as a biomedical and diagnostic tool,[^22^, ^29^, ^30^, ^31^] this new method might also contribute to further develop 2D‐IR for biomedical investigations. Performing 3D‐IR‐DOSY experiments with IR‐compatible microfluidics^[32]^ should make the sample injection in such experiments comparatively simple.

## Conclusion

Infrared diffusion‐ordered spectroscopy is a simple and cost‐effective way to simultaneously characterize size and chemical structure of molecules, molecular aggregates or complexes, or small particles. The two‐dimensional spectra are analogous to NMR‐DOSY spectra, and we hope that IR‐DOSY will become a useful addition to NMR‐DOSY. The separating power is comparable to that of NMR‐DOSY, and less than in typical chromatographic methods, but compared to chromatography it has the advantage that no prior knowledge of the chemical structure of the compounds in the sample is required. The separating power might be increased by adding elecrophoresis to separate the species in the sample solution. IR Spectroscopy generally provides less specific structural information than NMR, but there may be samples for which NMR is difficult to use, or practical reasons to prefer using an FTIR rather than an NMR spectrometer. Furthermore, 3D‐IR‐DOSY can provide conformational and ultrafast dynamical information about the size‐selected species that may be difficult to obtain using other methods.

In the experiments reported in this article, the quantitative analysis of the time‐ and frequency dependent data could be done in simple manner. However, for more complicated samples more advanced analysis methods might be necessary, and we think that the extensive framework of sophisticated data‐analysis methods already developed in NMR research[^4^, ^7^, ^33^, ^34^] will be of use for this purpose.

One can think of a number of interesting applications of IR‐DOSY (patent pending). Supramolecular complexes often contain carbonyl or other functional groups with intense IR bands, that can be valuable markers for complexation. Mixtures of such complexes with their monomeric building blocks or small oligomers may be difficult to characterize with conventional IR spectroscopy, but with IR‐DOSY the monomers and different complexes can be investigated separately. Another interesting application are protein aggregates and fibrils, where the (2D)IR spectrum provides conformation‐sensitivity[^35^, ^36^, ^37^] and the diffusion‐coefficient‐selectivity makes it possible to investigate monomers, oligomers, and fibrils, which typically coexist in a sample. Polymers and plastic nanoparticles constitute a third interesting category of samples. Because of the size‐selectivity and structure‐sensitivity, we think that IR‐DOSY can also find application in the pharmaceutical and biomedical lab. In the pharmaceutical context, IR‐DOSY has potential as a tool to detect and quantify the concentration of small molecules such as trifluoroacetic acid (used for deprotection), small amounts of which are often present in pharmaceutical products. In the biomedical context, 2DIR‐DOSY can be used to detect and structurally characterize low‐molecular weight species in human blood serum, for which the use of 2DIR spectroscopy as a diagnostic tool has already been demonstrated.^[30]^ In all cases, the (2D)IR spectrum provides structural information, and the diffusion coefficient provides valuable information about the size or size distribution of the molecules or molecular aggregates in the sample.^[16]^

## Conflict of interest

The authors declare no conflict of interest.

1

## Supporting information

As a service to our authors and readers, this journal provides supporting information supplied by the authors. Such materials are peer reviewed and may be re‐organized for online delivery, but are not copy‐edited or typeset. Technical support issues arising from supporting information (other than missing files) should be addressed to the authors.

Supporting Information

## Data Availability

The data that support the findings of this study are available from the corresponding author upon reasonable request.
