# Supplementary material for: Infrared Diffusion‐Ordered Spectroscopy Reveals Molecular Size and Structure
Source: Angew Chem Int Ed Engl. 2022 Dec 7;62(2):e202213424. doi: 10.1002/anie.202213424 (PMC10107201; doi:10.1002/anie.202213424)
Supplement: Supplementary file 1 — Supporting Information [file ANIE-62-0-s001.pdf]

Angewandte Chemie
Eine Zeitschrift der Gesellschaft Deutscher Chemiker

Supporting Information

Infrared Diffusion-Ordered Spectroscopy Reveals Molecular Size and Structure

G. Giubertoni, G. Rombouts, F. Caporaletti, A. Deblais, R. van Diest, J. N. H. Reek, D. Bonn, S. Woutersen**

Experimental Section

1. Methods

1.1. Experiments

Sample preparation Polyethylene oxide (PEG) (Sigma-Aldrich) with average Mw $\sim$ 900,000 was dissolved in D_2O solution at a concentration of 2% wt and was mixed using a roller shaker for at least 48 hours to obtain a transparent and homogeneous solution. Dialanine (Bachem) and acetone (Sigma-Aldrich) were dissolved to D_2O-PEG or D_2O at a concentration of 40 mg/ml and 4% (v/v). Bovine serum albumin or BSA (powder form, Sigma-Aldrich) and glucuronic acid (powder form, Sigma-Aldrich) were dissolved in D_2O-PEG solution at a concentration of 60 mg/ml and 40 mg/ml, respectively. We use D2O as a solvent since the structure-sensitive vibrations of the solutes absorb in the same infrared frequency region as the bending mode of H2O (around 1650 cm^{-1}).
IR-DOSY setup In all the experiments, solvent and mixture were injected through 1 mL syringes using a pump-syringe (Harvard, model 22 MA1 55-2226) at a flow rate of 25 μl/min to avoid turbulent flow (see SI). At this rate, the injection time through the channel (which has a volume of about 1 μL) is 2 s, which is negligible compared to the diffusion time. To perform FTIR-microscopy an IR-DOSY cell with a 1.2 mm wide channel was used, while for FTIR/2DIR experiments an IR-DOSY cell with a 4 mm wide channel was used.
FTIR-microscopy FTIR microscopy experiments were performed on a PerkinElmer Spotlight 400 system equipped with a 16-pixel MCT array detector. The spectra were acquired at 3 cm^{-1} spectral resolution, and a pixel step size of 6 or 25 μm to image the channel cross-section of the double mixture and triple mixture, and of 50 μm to image the full channel. **FTIR-spectroscopy** Each 1D-IR (FTIR) spectrum was an average of 32 scans, and measured between 600-7000 cm^{-1} with a 2 cm $^{-1}$ spectral resolution using a a Bruker Vertex 70. Time-dependent FTIR spectra were recorded every 90 s.
2DIR spectroscopy A detailed description of the setup used to measure the 2DIR spectra can be found in ref. 1. Briefly, pulses of wavelength 800 nm and with a 40 femtosecond duration are generated by using a Ti:sapphire oscillator, and further amplified by using a Ti:sapphire regenerative amplifier to obtain 800 nm pulses at 1 kHz repetition rate. These pulses are converted in an optical parametric amplifier to obtain mid-IR pulses ($\sim$20 μJ, $\sim$6100 nm) that has a spectral full width at half max (FWHM) of 150 cm^{-1}. The beam is split into a probe and reference beam (each 5%), and a pump beam (90%) that is aligned through a Fabry-Pérot interferometer. The pump and probe beams are overlapped in the sample in an $\sim$250-μm focus. The transmitted spectra of the probe (T) and reference (T_0) beams with pump on and off are recorded after dispersion by an Oriel MS260i spectrograph (Newport, Irvine, CA) onto a 2$\times$32-pixel mercury cadmium telluride (MCT) array. The probe spectrum is normalized to the reference spectrum to compensate for pulse-to-pulse energy fluctuations. The 2DIR signal is obtained by subtracting the probe absorptions in the presence and absence of the pump pulse.

1.2. Quantitative analysis

Time- and position-dependent concentration and absorption To analyze the time-dependent spectral data and convert it into a DOSY spectrum, we use numerical solutions to the diffusion equation. First we analyze the diffusion of a single compound from the solution-filled half of the cell into the solvent-filled half. Sufficiently far away from the entrance and exit of the channel, this diffusion process is effectively 1-dimensional. At $t = 0$, the spatial profile of the concentration is a step function, and the subsequent evolution of the position- and time-dependent concentration is described by the diffusion

equation. Specifically, for a channel of width L centered at $y = 0$, the diffusion equation for the time and position dependent concentration $c(y, t)$ of a particular species

$$\frac{\partial^2 c(y,t)}{\partial y^2} = D\frac{\partial c(y,t)}{\partial t} \tag{1}$$

with D the diffusion coefficient, and with initial concentration profile $c(y, 0) = 1$ for $-L/2 \leq y < 0$ and $c(y, 0) = 0$ for $0 \leq y \leq L/2$, and boundary condition $\partial c/\partial y = 0$ for $y = \pm L/2$ (no flux at the boundaries). Introducing dimensionless length $\xi = y/L$ and time $\tau = Dt/L^2$, the solution is $c(y, t) = C(y/L, Dt/L^2)$, where[2]

$$C(\xi,\tau) = \frac{1}{2} - \frac{2}{\pi}\sum_{n=0}^{\infty}\frac{\sin\left[\pi(2n+1)\xi\right] e^{-\pi^2(2n+1)^2\tau}}{2n+1}. \tag{2}$$

For $\tau \ll 1$ the sum converges slowly, and then it is more efficient to use the image-charge method[3] which gives

$$C(\xi,\tau) = \frac{1}{2} - \frac{1}{2}\sum_{n=-\infty}^{\infty}\mathrm{erf}\left(\frac{\xi+n}{2\sqrt{\tau}}\right), \tag{3}$$

where $\mathrm{erf}(x) = (2/\sqrt{\pi})\int_0^x e^{-t^2}dt$. To efficiently calculate $c(y, t)$, we use eq. (2) for $\tau > 0.2$ and eq. (3) for $\tau < 0.2$. Using the first two terms of eq. (2), and the terms $-3 \leq n \leq 3$ of eq. (3), we obtain a precision at $x = L/2$ of $< 10^{-8}$ all times. This describe the concentration (and hence absorption) profile of a single component (Fig.2C in the main text). When there are N species in the solution, with IR spectra $A_i(\nu)$ (with i the species number and ν the IR frequency), the total absorption at IR frequency ν, position y and time t is given by

$$A(\nu, y, t) = \sum_{i=1}^{N} A_i(\nu)C(y/L, D_i t/L^2), \tag{4}$$

where D_i is the diffusion coefficient of species i. This expression was used to fit the IR-image data of Fig.2 in the main text (see section "Least-square fitting procedures" for further details). In the 2D-IR-DOSY experiments, we sample the IR absorption in a spatial range close to the edge of the channel, selected using a slit. In this case we should integrate eq. (4) from $y = L/2 - w_0$ to $L/2$ (with w_0 the slit width), but numerical calculation shows that if the slit is not too wide (w_0 less than $\frac{1}{8}$ of the channel width L), the integral can be approximated to within less than 1% by the concentration at $L/2$, and so we fit the data in Figs.3 and 4 in the main text simply by

$$A(\nu, L/2, t) = \sum_{i=1}^{N} A_i(\nu)C(1/2, D_i t/L^2). \tag{5}$$

For the 3D-DOSY experiments, we proceed in a similar manner, except that in eq. (5) the species absorption spectra are now two-dimensional functions $A_i(\nu_{\mathrm{pump}}, \nu_{\mathrm{probe}})$.

Obtaining the IR-DOSY spectrum from the data To obtain DOSY spectra from the time- and frequency-resolved data, we use a procedure similar to equation (2) of Ref. 4. By least-squares fitting eq. 5 (or eq. 4 in the case of Fig. 2), we obtain diffusion constants D_i and their associated spectra $A_i(\nu)$ (see section "Least-square fitting procedure"). The 2D-DOSY spectrum $S(\nu, D)$ is obtained by multiplying the spectral amplitude $A_i(\nu)$ with the appropriate probability distribution for D_i:[4]

$$S(\nu, D) = \sum_{i=1}^{N} A_i(\nu)\frac{e^{-(D-D_i)/2\sigma_i^2}}{\sqrt{2\pi\sigma_i}}$$

where N is the number of species ($N = 2$ in Fig.2 and 3, $N = 3$ in Fig.4), and σ_i are the uncertainties in the diffusion coefficients obtained from the least-squares fits.

2. Reynolds number in a Hele-Shaw channel

Since $\alpha = \frac{L}{H} >> 1$, where L and H are the width and height of the channel, we calculate the Reynolds number Re in a Hele-Shaw channel by using the following equation[5]:

$$Re = \frac{v_o H}{\nu} \cdot \frac{1}{\alpha} \tag{6}$$

where v_0 is the flow speed and ν the kinematic viscosity. Since the flow rate is defined as $Q = v_O \cdot LH$, we can write eq.6 as:

$$Re = \frac{Q}{\nu L} \cdot \frac{1}{\alpha} \tag{7}$$

By using $L = 1 \cdot 10^{-3}[m]$,$H = 50 \cdot 10^{-6}[m]$, $\nu = 10^{-6}[m^2/s]$ and $Q = 0.42 \cdot 10^{-9}[m^3/s]$, we obtain that $Re \sim 0.02$, indicating that in the IR-DOSY experiment the flow is laminar. Note that at $t = 0$ we stop the flow, and all spectra are recorded in absence of flow.

3. IR-DOSY of a double mixture in pure D_2O

We perform IR-DOSY on a solution containing acetone (4 % vv) and dialanine (40 mg/ml) dissolved in D_2O. We first perform IR-microscopy to check whether the interface between the mixture and the solvent was centered at half of the channel. Fig. S1 shows IR-microscope images while infusing and after stopping the flow, where we can observe that in both case the interface is at half of the channel. The colorbar represents the absorption intensity of the acetone peak at 1695 cm^{-1}. Fig. S2A shows the absorption spectra as a function of time of the double mixture. At 5 and 10 minutes, we clearly see the absorption peak of the acetone at 1695 cm^{-1}, while the bands of the dialanine at 1650 and 1595 cm^{-1} appear later. Fig. S2B–C show the absorption signals as a function of time at the peak-frequencies of the two compounds. By fitting the data, we find a diffusion coefficient of $6.5 \pm 1 \cdot 10^{-6}$ cm^2/s for acetone, and $4 \pm 0.4 \cdot 10^{-6}$ cm^2/s for dialanine. We used these values to globally fit the time-dependent spectra in the frequency region between 1350 and 1750 cm^{-1}. The IR-DOSY spectrum is shown in Fig. S3. The higher diffusion coefficients with respect to the double mixture dissolved in D_2O-PEG is due to the absence of PEG in this experiment.

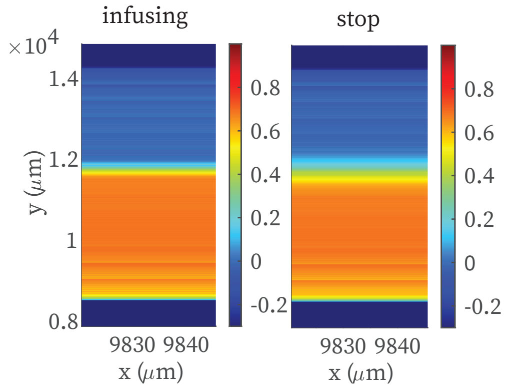

Fig S 1 – IR-microscope images of the channel cross-section while infusing (left) and immediately after stopping the infusion (right) the solution (bottom half) and solvent. The solvent is pure D_2O. Color bar: absorption at the frequency of the acetone (1695 cm^{-1}).

4. IR-microscopy-imaging of a triple mixture in D_2O-PEG(2% wt)

Fig. S4 shows the spatial distributions of the acetone, glucuronic acid and BSA absorption across the channel at different times. These distributions can be well described using the solution of the diffusion equation (see Methods section in main text for the mathematical details), and from least-squares fits (shown as the curves in Fig. S4) we obtain the respective diffusion coefficients. We can use these diffusion coefficients to globally fit the entire set of time-dependent absorption profiles in the entire 1550–1750 cm^{-1} frequency range, where we keep the diffusion constants of the three compounds fixed to the values obtained at their peak-absorption frequencies, and treat the amplitudes of acetone, glucuronic acid and BSA at each frequency as fit parameters. In this way we obtain the IR-DOSY spectrum shown in Fig.4 in the main text.

5. Conventional infrared spectra of the component solutions

Fig. S5 shows the infrared spectrum of acetone at a concentration of 5 wt% dissolved in D_2O. We observe one peak absorbing one at 1690 cm^{-1}, and two broader and congested bands centered 1450 and at 1380 cm^{-1}. The first is assigned to the C=O of the acetone, while the last two to side-chain modes. Fig. S6 shows the infrared spectrum of dialanine at a concentration of 20 mg/ml dissolved in D_2O. In the amide I region, we observe two peaks absorbing one at 1650 cm^{-1} (amide I) and one at 1590 cm^{-1} (-COO^-, anti-symmetric stretching), while a series of peaks in the region between 1350 and 1500 cm^{-1}. In this second spectral window, we observe two more intense bands absorbing one at 1490 cm^{-1} (amide II) and one at 1400 cm^{-1} (-COO^-, symmetric stretching). Fig. S7 shows the infrared spectrum of BSA at a concentration of 30 mg/ml dissolved in D_2O. We observe three peaks absorbing one at 1650 cm^{-1}, 1550 cm^{-1} and at 1580 cm^{-1}. This last can be assigned to the anti-symmetric stretching of COO^--side groups[6].

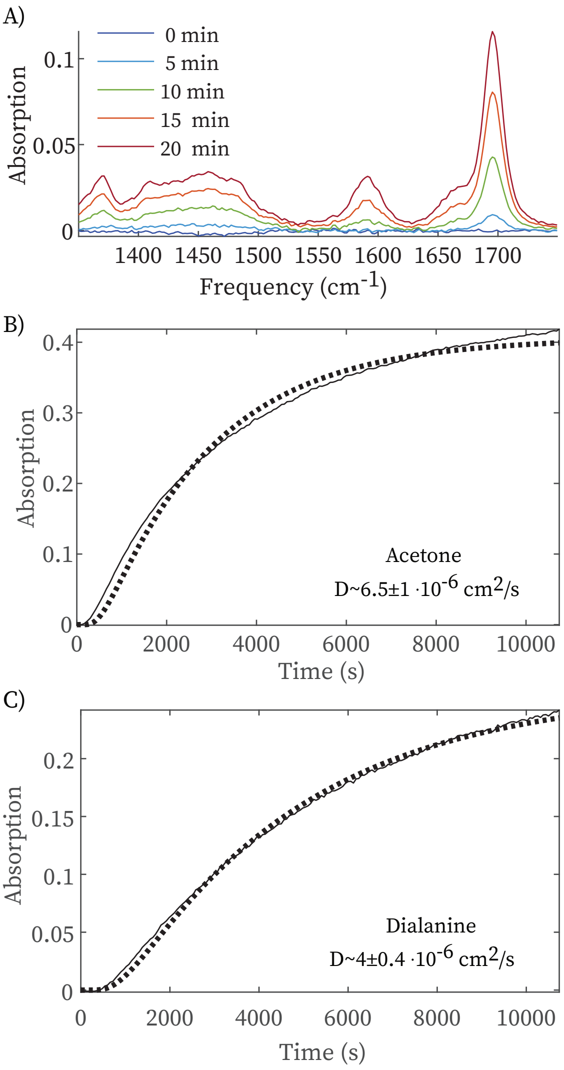

Fig S 2 – (A) Absorption spectra as a function of time for a solution containing acetone (4 % wt) and dialanine (40 mg/ml) dissolved in pure D_2O. (B)-(C) Absorption (continuous line) and fit (dashed line) as a function of time of for acetone and dialanine, respectively.

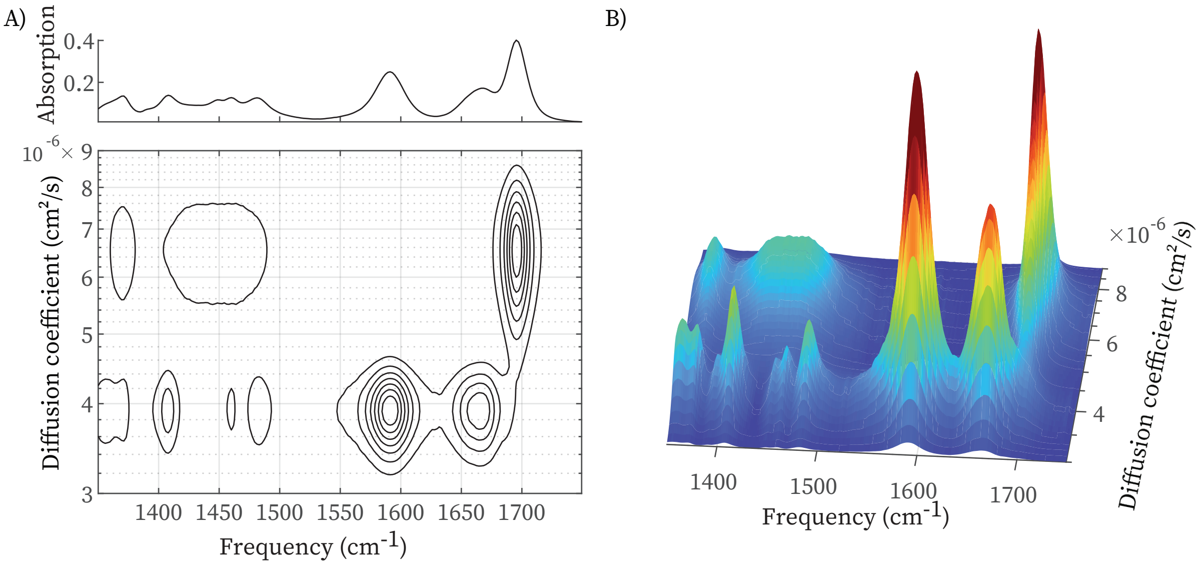

Fig S 3 – IR-DOSY spectrum obtained for a mixture of acetone and dialanine dissolved in pure D_2O, as contour (A) and surface (B) graphs.

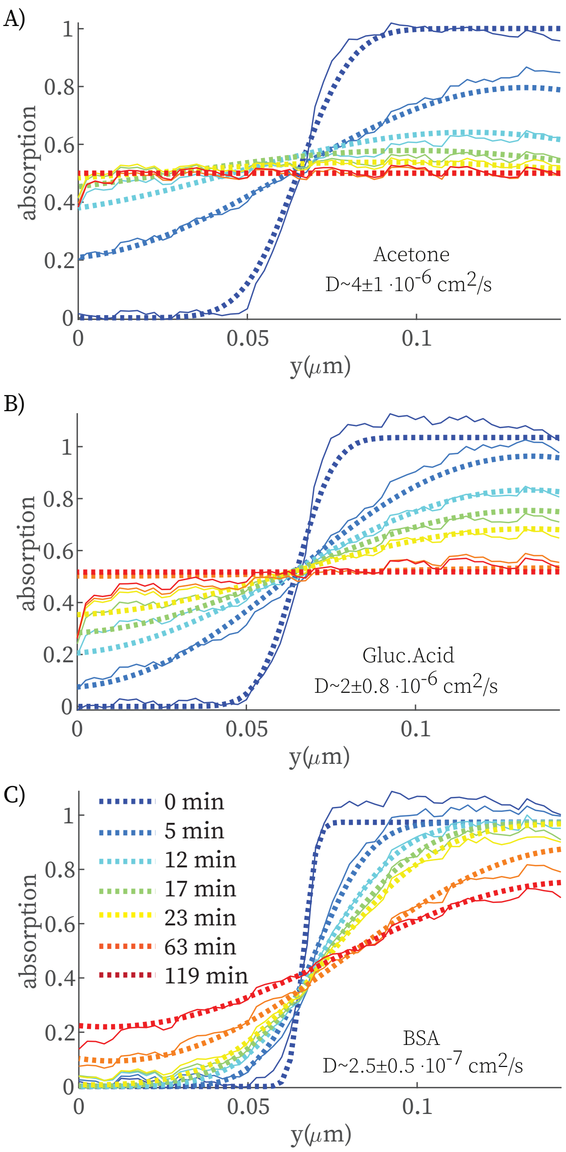

Fig S 4 – A), B) and C) Spatial distributions of the acetone, glucuronic acid and BSA IR absorption at different time delays after stopping the flow, respectively. The dashed curves are least-squares fits of the numerical solution of the diffusion equation.

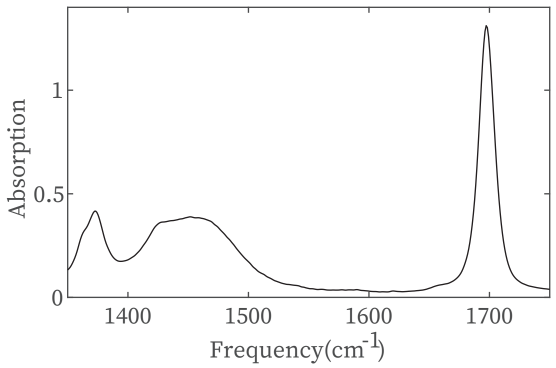

Fig S 5 – Infrared spectrum of a D_2O solution of acetone at a concentration of 5 % wt.

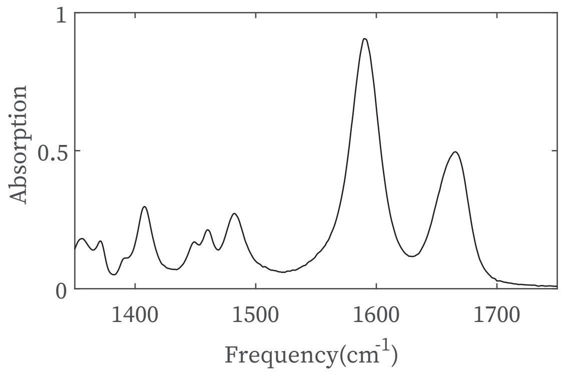

Fig S 6 – Infrared spectrum of a heavy water solution of dialanine at a concentration of 20 mg/ml.

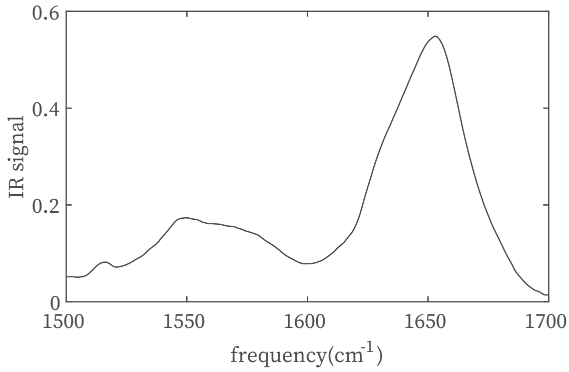

Fig S 7 – Infrared spectrum of a heavy water solution of BSA at a concentration of 30 mg/ml.

6. Fit of the time-dependent 2D-IR signals

To extract the diffusion coefficients for BSA and acetone, we fit the time-dependency of 2DIR signal at the specific $(\nu_{\mathrm{pump}}, \nu_{\mathrm{probe}})$ of their respective diagonal negative peaks. These positions are chosen by taking the pump/probe frequencies where we have the minimum diagonal signals in the last recorded 2DIR spectrum. See Figure S8 for the data and the fit. The data are plotted positive for convenience.

7. Least-squares fitting procedures

7.1. IR-DOSY microscopy

In case of measurements performed by using an IR microscope, infrared images on the x-y plane are collected at a specific z-position, where x and y represent the length and width of the channel, respectively. Absorption data $\alpha^{\mathrm{MicroIR}}$ are provided as a function of frequency, ν, time t, y and x, written as matrix $K \times M \times Y \times X$, where K is the number of frequencies, M the number of times, Y number of y-pixels and X number of x-pixels. Infrared images are averaged over the x direction so to obtain for every frequency $\overline{\nu}$, absorption profiles as a function of time and y, $\alpha^{\mathrm{MicroIR}}(\overline{\nu}, t, y)$. These can be described by the model function (see Methods section main text):

$$f(t,y) = A\left(\frac{1}{2} - \frac{2}{\pi}\sum_{n=0}^{21}\frac{\sin\left[\pi(2n+1)\frac{(y+y^{\mathrm{off}}(t))}{L}\right]e^{-\pi^2(2n+1)^2 D(t+t_{\mathrm{off}})}}{2n+1}\right) \tag{8}$$

where m is the number of time delays, A is the amplitude at a frequency $\overline{\nu}$, y_i^{off} is the offset along the y, D the diffusion coefficient at a frequency $\overline{\nu}$, t_{off} is the time offset due to the initialization of the instrument before recording every IR image ($\sim$20 s) and L is the height of the cross-section ($\sim$0.13 cm). In our fitting analysis, we first fit the absorption profiles at specific frequencies, where the species in the mixture absorb. In this case, A, D, y_i^{off} are global parameters that can be optimized by a fitting routine (*lscurvefit*, MatLab). After we obtain the diffusion coefficients $\overline{D_j}$ with $j = 1 \ldots$(# of species), we use them as fixed input parameters to globally fit the absorption profiles at every frequency in the selected spectral region by using the model function

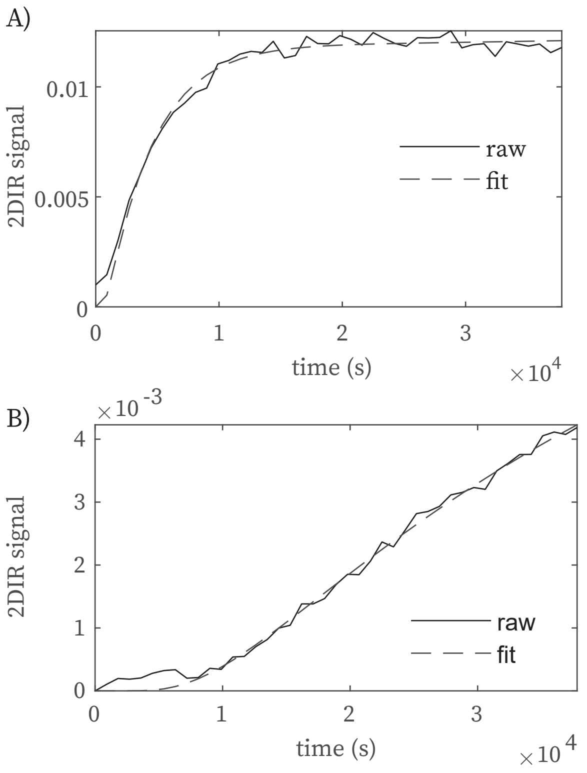

Fig S 8 – A) and B) raw and fit data of the diagonal negative signals at the pump and probe absorption frequencies of the acetone and BSA, respectively.

$$f(\nu_i, t, y) = \sum_{j=1}^{3} A_j(\nu_i) \left(\frac{1}{2} - \frac{2}{\pi} \sum_{n=0}^{21} \frac{\sin\left[\pi(2n+1)\frac{(y+y^{\text{off}})}{L}\right] e^{-\pi^2(2n+1)^2 \overline{D}_j (t+t_{\text{off}})}}{2n+1} \right) \tag{9}$$

where the spectral amplitudes $A_j(\nu_i)$ are fit parameters.

7.2. IR-DOSY

In case of measurements performed by using FTIR spectrometer, absorption spectra are collected at specific time intervals. Time-dependent absorption data α^{IR} are provided as a function of frequency, ν and time t, written as a matrix $K \times M$, where K is the number of frequency and M the number of times. To fit these data, we first define a new variable

$$t_D = D\frac{t + t_{\text{offset}}}{L^2} \tag{10}$$

where t_{offset} is a time offset due to possible delay in the instrument initiation, and L is the width of the channel. In this case, $t_{\text{offset}} = 0$ s, and $L = 0.4$ cm. For every frequency $\overline{\nu}$, the time-dependent absorption signal, $\alpha^{IR}(\overline{\nu}, t_D)$ can be described by the following model function (see Experimental section of main text):

$$f(t) = \begin{cases} A\left(\frac{1}{2} - \frac{1}{2}\sum_{n=-3}^{3} \text{erf}\left[\frac{n}{2\sqrt{t_D}}\right]\right) & \text{if } t_D < 0.2 \\ A\left(\frac{1}{2} - \frac{2}{\pi}e^{-t_D\pi^2}\right) & \text{if } t_D > 0.2 \end{cases} \tag{11}$$

The formula for $t_D > 0.2$ in eq. (6) is obtained by taking the first two terms of eq.(4) with $y = L/2$. In our fitting analysis, we first fit the absorption profiles at specific frequencies, where the species in the mixture absorb. In this case, A and D are global parameters that can be optimized by a fitting routine (*lscurvefit*, MatLab). After we obtain the diffusion coefficients $\overline{D_j}$ with $j = 1 \ldots$(# of species), we use them as fixed input parameters to globally fit the absorption spectra in the selected region by using the following model function:

$$f(t,\nu) = \begin{cases} \sum_j A_j(\nu)\left(\frac{1}{2} - \frac{1}{2}\sum_{n=-3}^{3} \text{erf}\left[\frac{n}{2\sqrt{t_{D_j}}}\right]\right) & \text{if } t_{D_j} < 0.2 \\ \sum_j A_j(\nu)\left(\frac{1}{2} - \frac{2}{\pi}e^{-t_{D_j}\pi^2}\right) & \text{if } t_{D_j} > 0.2 \end{cases} \tag{12}$$

where the species spectral amplitudes $A_j(\nu)$ are global fit parameters.

7.3. 3D-IR-DOSY

In case of measurements performed by using 2DIR, absorption spectra are collected at specific time intervals as a function of pump (ν_{pump}) and probe frequency (ν_{probe}). Time-dependent absorption data α^{2DIR} are provided as a function of ν_{pump},ν_{probe} and time t, written as matrix $K \times S \times M$, where K is the number of pump frequency, S the number of probe frequency and M the number of times. Similarly to IR-DOSY, we use eq. 11 to extract the diffusion coefficients for the species present in the mixtures by fitting first the time-dependent 2D-signal at the frequency positions of the diagonal negative peaks (see Fig.8). After we obtain the diffusion coefficients $\overline{D_j}$ with $j = 1\ldots$(# of species), we use them as fixed input parameters to globally fit the 2D-signal, $\alpha^{\mathrm{2DIR}}(\nu_{\mathrm{pump}}, \nu_{\mathrm{probe}}, t)$, by using the following model function:

$$f(\nu_{\mathrm{pump}}, \nu_{\mathrm{probe}}, t) = \begin{cases} \sum_j A_j(\nu_{pump}, \nu_{probe}) \left(\frac{1}{2} - \frac{1}{2} \sum_{n=-3}^{3} \mathrm{erf}\left[\frac{n}{2\sqrt{t_{D_j}}} \right] \right) & \text{if } t_{D_j} < 0.2 \\ \sum_j A_j(\nu_{pump}, \nu_{probe}) \left(\frac{1}{2} - \frac{2}{\pi} e^{-t_{D_j}\pi^2} \right) & \text{if } t_{D_j} > 0.2 \end{cases} \tag{13}$$

where the species spectral amplitudes $A_j(\nu_{pump}, \nu_{probe})$ are global fit parameters.

References

[1] A. Huerta-Viga, D. J. Shaw, S. Woutersen, *Journal of Physical Chemistry B* **2010**, *114*, 15212.
[2] R. Ghez, *A Primer of Diffusion Problems*, Wiley, New York **1988**.
[3] V. Balakrishnan, *Elements of Nonequilibrium Statistical Mechanics*, Springer **2021**.
[4] G. A. Morris, Diffusion-Ordered Spectroscopy, in R. K. Harris, R. E. Wasylishen (Editors), *Encyclopedia of Magnetic Resonance*, Wiley, Chichester, UK **2009**.
[5] P. Oswald, *Rheophysics : the deformation and flow of matter*, Cambridge University Press **2014**.
[6] J. Grdadolnik, Y. Maréchal, *Biopolymers* **2001**, *62*, 40.
